# Pattern and risk factors of local recurrence after nephroureterectomy for upper tract urothelial carcinoma

**DOI:** 10.1186/s12957-020-01877-w

**Published:** 2020-05-30

**Authors:** Xiaoying Li, Ming Cui, Xiaobin Gu, Dong Fang, Hongzhen Li, Shangbin Qin, Kunlin Yang, Tianzhao Zhu, Xuesong Li, Liqun Zhou, Xian-Shu Gao, Dian Wang

**Affiliations:** 1 Department of Radiation Oncology, Peking University First Hospital, Peking University, Beijing, China; 2 Department of Urology, Peking University First Hospital, Peking University, Beijing, China; 3 Department of Medical Imaging, Peking University First Hospital, Beijing, China; 4 Department of Radiation Oncology, Rush University Medical Center , Chicago, USA

**Keywords:** Upper tract urothelial carcinoma, Local recurrence, Risk factor, Primary tumor location

## Abstract

**Purpose:**

This study aims to identify predictive local recurrence risk factors and site-specific local recurrence pattern of upper tract urothelial carcinoma (UTUC) with different primary tumor locations.

**Methods:**

Three hundred and eighty-nine UTUC patients with radical nephroureterectomy were included in this study. Univariate and multivariate Cox proportional hazards regressions were performed to measure the risk of local recurrence. We also mapped the position of local recurrence sites stratified by primary tumor locations.

**Results:**

A total of 73 patients (18.7%) developed local recurrence within a median follow-up of 41 months (range, 3-80 months). For patients with local recurrence, the median interval of local recurrence was 9 months. Ureter tumor, multifocality, T stage, G grade, lymph node metastasis (LNM), lymph node dissection (LND), and lymph vascular invasion (LVI) were all significantly associated with increased local recurrence by univariable analyses (*P* < 0.05). Only multifocality, T3–4, G3, and LNM remained independent predictors of increased local recurrence by multivariable analyses. Adjuvant radiotherapy could reduce the local recurrence (HR = 0.177; 95% CI 0.064-0.493, *P* = 0.001). Patients with local recurrence had poorer cancer-specific survival (4-year cancer-specific survival rate 36 ± 7.5% vs 88.4 ± 2.2%, *P* = 0.000). We evaluated local recurrence pattern stratified by tumor locations. Para-aortic lymph node region was the most common recurrence area for all the patients. Left-sided UTUC had more than 70% recurrent lymph nodes in the left para-aortic region (LPA). For right-sided UTUC patients, recurrent para-aortic lymph nodes distributed in the LPA (33.3%), aortocaval (AC) (41.5%), and right paracaval (RPC) (25.2%) regions. Recurrence in the internal and external iliac regions was only found in the distal ureter group (*P* < 0.05). Renal pelvic fossa recurrence was only found in renal pelvic tumor (22.2%, *P* = 0.007). The ureter tumor bed recurrence rate was higher for ureter patients (*P* = 0.001).

**Conclusions:**

Multifocality, T3–4, G3, and LNM are predictors of higher local recurrence rate of UTUC. Adjuvant radiotherapy can reduce local recurrence rate. Local recurrence patterns are different according to primary tumor locations.

## Introduction

UTUC is a relatively uncommon urinary malignant tumor and represents approximately 5% of all urothelial malignancies in the USA [1]. The incidence rate of UTUC is unusually higher in Asia. The intake of arsenic-contaminated water and the consumption of Chinese herbs are claimed to be one of the important etiologic factors for UTUC in China [2–6].

Radical nephroureterectomy (RNU) is the mainstay treatment for UTUC [7]. Several retrospective reviews have reported a locoregional failure rate varied from 6.2 to 65% in UTUC patients who underwent RNU [6, 8–13]. Factors such as multiple tumor focal, incomplete surgery, LVI, tumor grade, and T stage were found associated with local recurrence [9, 14, 15]. Template-based and complete LND was not routinely performed, which may also cause the high local recurrence rate in high-risk patients [16]. Metastasis patterns of UTUC with different primary tumor locations were different [17]. However, there is no study about the site-specific local recurrence pattern of UTUC. The role of adjuvant radiotherapy on UTUC is also not well defined [18].

Thus, in our study, we aimed to identify predictive factors of local recurrence and the effect of adjuvant radiotherapy after radical nephroureterectomy. In this study, we also analyzed the local recurrence pattern of UTUC with different primary tumor locations.

## Methods

### Patient and treatments

A total of 506 patients with UTUC who underwent radical nephroureterectomy from July 2010 to July 2015 in the Peking University First Hospital were reviewed. We excluded 21 patients who had a segmental resection. We also excluded 30 patients who had adjuvant chemotherapy. Sixty-six patients were lost of follow-up. Finally, a total of 389 patients were eligible for analysis. All patients underwent radical nephroureterectomy with bladder cuff resection. Laparoscopic nephroureterectomy was the standard treatment in our hospital. Open nephroureterectomy was only used for patients with wild LND. Transurethral resection of bladder tumor (TURBT) treatment was used for patients with concomitant bladder tumor during the surgery. LND was conducted when LNM was suspected in the preoperative evaluation or enlarged lymph nodes were found during surgery. The extent of our standard lymphadenectomy was the ipsilateral side of the great vessels and renal hilum for tumors of the renal pelvis, upper ureter, and middle ureter and the ipsilateral pelvic lymph nodes for tumors of the lower ureter. In some patients, lymphadenectomy was extended to the retrocaval and/or aortocaval nodes [12].

Histological diagnosis was determined according to the 2004 World Health Organization (WHO) classifications. Tumors were pathologically staged according to the Eighth American Joint Committee on Cancer (AJCC) staging system. Two hundred and seventy-eight patients (71.5%) were pure transitional cell carcinoma; 19 patients (4.9%) were transitional cell carcinoma with adenocarcinoma differentiation; 42 patients (10.8%) were transitional cell carcinoma with squamous cell differentiation; 29 patients (7.5%) were transitional cell carcinoma with necrosis; 6 patients (1.5%) were transitional cell carcinoma with sarcoma; 15 patients (3.9%) were other tumor types. Tumor locations were classified into two groups: renal pelvic tumor and ureter tumor. The ureter tumor group was classified into three parts: proximal ureter tumor defined as above the upper border of the sacrum, middle ureter tumor defined as from the upper to the lower border of the sacrum, and distal ureter defined as below the lower border of the sacrum [19]. Multiple tumors were defined as two or more separate tumors (not contiguous extension) within the upper urinary tract. When tumors were involved in multiple sites, primary tumor location was defined by the highest T stage and/or grade.

Inclusion criteria of the study were as follows: (1) pathologic diagnosis of urothelial carcinoma of renal pelvic or ureter, (2) pathological stage T1-4aN0-2 M0, (3) no primary tumor was left by positron emission tomography (PET) or CT 1 month after surgery, and (4) regular follow-up at our center. The exclusion criteria were as follows: (1) patients with previous or sequential second primary cancers (except for bladder tumor), (2) non-radical nephroureterectomy, and (3) patients with adjuvant chemotherapy.

Patients with adverse risk factors, such as T3–4, G3, or positive lymph node, were suggested to use adjuvant radiotherapy. Finally, 73 of them (18.8%) received adjuvant radiotherapy. Adjuvant radiotherapy was performed using volumetric-modulated Arc radiotherapy (VMAT) techniques with 6-MV photons. The clinical target volume included the renal fossa, course of the ureter, abdominal, and pelvic lymph nodes. The median radiation dose was 50 Gy/25 fractions.

All the patients had regular follow-up every 3 months for the first 2 years after surgery, every 6 months in the next 3 years, and annually thereafter. Evaluations included a history, physical examination, blood test, urinary cytology, chest radiograph, cystoscopy, and ultrasonograms or CT/magnetic resonance imaging (MRI) scans of the abdomen.

### Patterns of locoregional failure

Locoregional recurrence was defined as lymph node relapse or surgical bed recurrence within the retroperitoneal and/or pelvis field. Diagnostic criteria for locoregional recurrence included the following: new adenopathy found by CT, MRI, or PET. The adenopathy definition is defined by a short axis diameter of 1 cm or larger in CT and/or MRI images [20–22]. The adenopathy definition is defined by PET/CT visualization of hypermetabolic activity regardless of size. The first locoregional recurrence was recorded in this study for analysis. The confluent lymph nodes were counted as one nodal recurrence. The adenopathy was reviewed and confirmed by two radiologists and were often recommended for pathologic confirmation via CT or ultrasound-guided biopsy. Recurrences in the lung, liver, bone, and other organs were categorized as distant metastasis. Distant lymph nodes were defined as those that occurred outside the abdomen and pelvis. Bladder relapse was not defined as a local recurrence in our study.

### Three-dimensional (3D) image rendering

All CT images were reconstructed to be a 3D anatomy model including the blood vessels. Positive lymphadenopathy was contoured and standardized into a 1-cm-diameter circle. Then, the distance from the volumetric center of each lymph node to vascular bifurcation was documented in each patient with lymph node recurrence. A map of nodal recurrence consists of para-aortic, common iliac, external iliac, and internal iliac regions [23]. Para-aortic lymph nodes are further divided into three subgroups: the left of the aorta (left para-aortic or LPA), between the aorta and inferior vena cava (aortocaval or AC), or to the right of the inferior vena cava (right paracaval or RPC) [24]. We plotted all the locoregional recurrence lymph nodes on the CT image of a patient according to their relative positions to the vessels and constructed a three-dimensional local recurrence map.

### Statistics

Patient characteristics were compared using either the Mann-Whitney *U* test or the chi-square test (or the Fisher exact test for smaller cell counts) for continuous or categorical variables, respectively. We used the Kaplan-Meier to estimate cancer-specific survival, and the log-rank test was applied to compare survival curves. Variables identified as significant by the univariate analysis were considered for the multivariate analysis. Univariate and multivariate Cox proportional hazards regression analyses were performed to determine the association between risk factors and local recurrence. We used the Statistical Package for Social Sciences (SPSS, version 22.0) to analyze all data. All tests were two-sided, and *P* values less than 0.05 were considered significant.

## Results

A total of 389 patients (Table 1) were eligible for analysis. Seventy-three patients (18.7%) had locoregional recurrence with a median follow-up time 41 months (3-80 months). The median time from surgery to local relapse was 39 months (range 1-80 m) for all the patients. Among the patients with locoregional recurrence, median time from surgery to local relapse was 9 months, and 27 patients (37.0% of 73 patients) also had distant metastasis at the same time.

**Table 1:** Patient clinical and pathological characteristic

Characteristics	All patients (%)	Patients with local recurrence (%)	Patients without local recurrence (%)	*P* value
Patient number	389	73 (18.8)	316 (81.2)	
Median age (range)	69 (35-88)	70 (37-85)	69 (35-88)	
Age group				0.620
< 70	202 (51.9)	36 (49.3)	166 (52.5)	
≥ 70	187 (48.1)	37 (50.7)	150 (47.5)	
Gender				0.727
Male	190 (48.8)	37 (50.7)	153 (48.4)	
Female	199 (51.2)	36 (49.3)	163 (51.6)	
Site				0.393
Left	185 (47.6)	38 (52.1)	147 (46.5)	
Right	204 (52.4)	35 (47.9)	169 (53.5)	
Location				0.042
Pelvic	212 (54.5)	32 (43.8)	180 (57.0)	
Ureter	177 (45.5)	41 (56.2)	136 (43.0)	
Concomitant bladder tumor				0.192
Yes	42 (10.8)	11 (15.1)	31 (9.8)	
No	347 (89.2)	62 (84.9)	285 (90.2)	
Tumor type				0.599
Complete TCC	278 (71.5)	54 (74.0)	224 (70.9)	
TCC with other differentiation	111 (28.5)	19 (26.0)	92 (29.1)	
Surgical procedure				0.088
Laparoscopic nephroureterectomy	326 (83.8)	57 (78.1)	269 (85.1)	
Open nephroureterectomy	63 (16.2)	16 (21.9)	47 (14.9)	
Lymph vascular invasion				0.009
Yes	58 (14.9)	18 (24.7)	40 (12.7)	
No	331 (85.1)	55 (75.3)	276 (87.3)	
Tumor grade				0.000
G1	6 (1.6)	0	6 (1.9)	
G2	209 (54.4)	17 (23.6)	192 (61.5)	
G3	169 (44.0)	55 (76.4)	114 (36.5)	
Pathological stage				0.000
T1	125 (32.6)	9 (12.3)	116 (36.7)	
T2	121 (31.5)	15 (20.5)	105 (33.3)	
T3	129 (33.6)	48 (65.8)	87 (27.5)	
T4	9 (2.3)	1 (1.4)	8 (2.5)	
Lymph node detection				0.000
No	316 (81.2)	48 (65.8)	268 (84.8)	
Yes	73 (18.8)	25 (34.2)	48 (15.2)	
Lymph node involvement				0.000
Nx	316 (81.2)	48 (65.8)	268 (84.8)	
N0	43 (11.1)	9 (12.3)	34 (10.8)	
N1	11 (2.8)	4 (5.5)	7 (2.2)	
N2	19 (4.9)	12 (16.4)	7 (2.2)	
Multifocality				0.013
No	321 (82.5)	53 (72.6)	268 (84.8)	
Yes	68 (17.5)	20 (27.4)	48 (15.2)	
Margin				0.189
Positive margin	12 (3.1)	4 (5.5)	8 (2.5)	
Negative margin	377 (96.9)	69 (94.5)	308 (97.5)	
Adjuvant radiotherapy				0.014
Yes	57 (14.7)	4 (5.5)	53 (16.8)	
No	332 (85.3)	69 (94.5)	263 (83.2)	

The clinical characteristics, stratified according to the presence of local recurrence, are shown in Table 1. In our study, 83.8% patients used laparoscopic nephroureterectomy. Only 16.2% patients used open nephroureterectomy. Although open nephroureterectomy had a higher local recurrence rate (25.39%), no significant difference was found in different surgical procedures (*P* > 0.05). A total of 73 patients (18.8%) had LND in our study. The median lymph node number removed was 4 (1-36). Local recurrence rate was higher in patients with LND (34.2% vs 15.2%). This may be because LND was only conducted when LNM was suspected in the preoperative evaluation. We can see complete transitional cell carcinoma and TCC with other differentiation had similar local recurrence rate (*P* > 0.005). On univariable analyses (Table 2), multifocality, T3–4, G3, LVI, LNM, LND, and ureter tumor were all significantly associated with increased local recurrence (*P* < 0.05). On multivariable analyses, only multifocality (HR = 2.116; 95% CI 1.246-3.593, *P* = 0.006), T3–4 (HR = 2.805; 95% CI 1.588-4.957, *P* = 0.000), G3 (HR = 2.991; 95% CI 1.659-5.393, *P* = 0.000), and LNM (HR = 1.925; 95% CI 1.053-3.521, *P* = 0.033) remained independent predictors of increased local recurrence.

**Table 2:** Univariable and multivariable Cox regression analyses predicting local recurrence in 389 patients who underwent a radical nephroureterectomy for upper tract urothelial carcinoma

Variables	Univariate analyses			Multivariate analyses		
	HR	95% CI	*P* value	HR	95% CI	*P* value
Age > 70 years	1.125	0.771-1.78	0.615			
Gender						
Male	1.121	0.708-1.775	0.626			
Female	1					
Site						
Left	1.213	0.766-1.92	0.409			
Right	1					
Location						
Pelvic	0.625	0.394-0.993	0.047	0.703	0.427-1.155	0.164
Ureter	1					
Focality						
Multifocality	2.005	1.226-3.446	0.006	2.116	1.246-3.593	0.006
Unifocality	1					
Tumor type						
TCC with other differentiation	0.921	0.546-1.555	0.759			
Complete TCC	1					
T stage						
T3–4	4.153	2.554-6.78	0.000	2.805	1.588-4.957	0.000
T1–2	1					
Tumor grade						
G3	4.674	2.744-7.963	0.000	2.991	1.659-5.393	0.000
G1–2	1					
LND						
Yes	2.802	1.727-4.548	0.000	1.129	0.547-2.334	0.742
No	1					
Surgical procedure						
Laparoscopic nephroureterectomy	0.678	0.389-1.181	0.678			
Open nephroureterectomy	1					
LNM						
Yes	5.017	2.872-2.163	0.000	1.925	1.053-3.521	0.033
No	1					
LVI						
Yes	2.12	1.245-3.612	0.006	1.202	0.671-2.151	0.536
No	1					
Concomitant bladder tumor						
Yes	1.602	0.843-3.042	0.150			
No	1					
Positive margin						
Yes	2.053	0.749-5.63	0.162			
No	1					
Adjuvant radiotherapy						
Yes	0.313	0.114-0.861	0.024	0.177	0.064-0.493	0.001
No	1					

Adjuvant radiotherapy could significantly reduce local recurrence rate of UTUC on multivariable analyses (HR = 0.177; 95% CI 0.064-0.493, *P* = 0.001) (Table 2). Only 4 patients in the adjuvant radiotherapy group had local recurrence. Two of the four patients had lymph node recurrence out of radiation field.

**Fig. 1:**
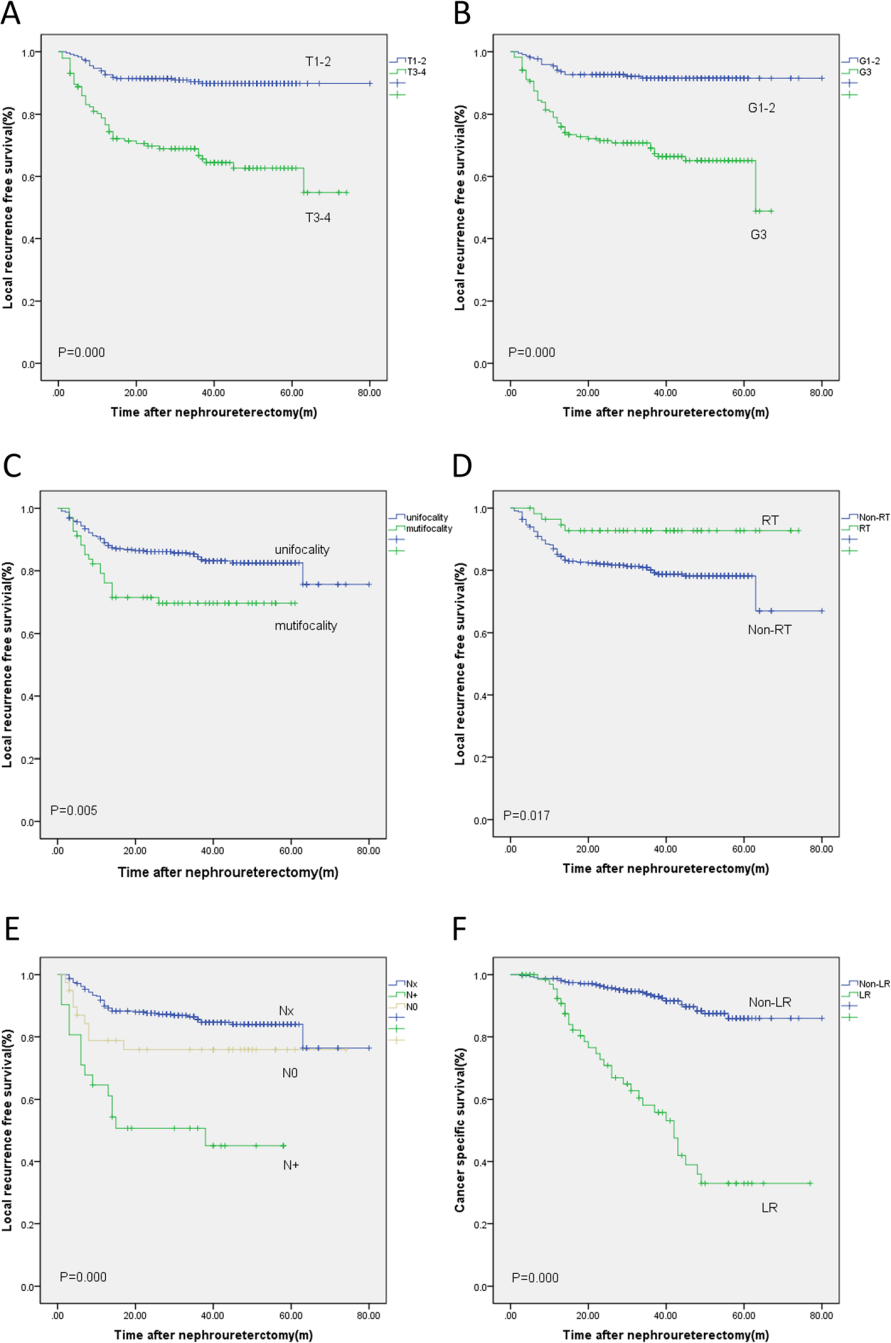
The Kaplan-Meier survival curves of local recurrence-free survival stratified by T stage (**a**), G grade (**b**), multifocality (**c**); radiation therapy (RT) (**d**); lymph node status (**e**); cancer specific-free survival stratified by local recurrence (LR) (**f**)

Patients with isolated local recurrence received salvage radiotherapy. Chemotherapy was used for patients with both local recurrence and distant metastasis. Ninety-nine patients (25.45%) had bladder recurrence during the follow-up time in our study. Patients with bladder recurrence received TURBT or radical cystectomy. Patients with local recurrence had poorer cancer-specific survival. The 4-year cancer-specific survival (CSS) rate was only 36 ± 7.5 % in local recurrence patients compared with 88.4 ± 2.2% in non-local recurrence patients (*P* = 0.000, Fig. 1f).

We also analyzed site-specific local recurrence pattern with different primary tumor locations for patients without radiation therapy. Six patients who could not provide local recurrence image information were excluded. We stratified the other 63 local recurrent patients into four groups by primary tumor location. Twenty-seven patients had renal pelvic tumors, 3 had proximal ureter tumors, 9 had middle ureter tumors, and 24 had distal ureter tumors. Table 3 shows the local recurrence patterns of UTUC patients with different primary locations. Lymphatic recurrence was the main local recurrence type. The para-aortic lymph node region was the most common recurrence area (73% of all the patients). Common iliac lymph node region was the second common recurrence region for all the patients (31.7% of all the patients). Recurrences in the internal and external iliac regions were only found in distal ureter tumor (16.7% of distal ureter tumor, *P* = 0.018). None of the patients in the ureter tumor group had renal fossa recurrence. Renal pelvic fossa recurrence only occurred in the renal pelvic tumor (22.2% of renal pelvic tumor, *P* = 0.007). The ureter tumor bed recurrence rate was higher in ureter tumor (45.8% in distal ureter, 22.2% in middle ureter, 66.7% in proximal ureter, *P* = 0.001).

**Table 3:** Local recurrence rate of UTUC with different primary tumor locations

Local relapse region	All patients (*N* = 63)	Renal pelvic (*N* = 27)	Proximal ureter (*N* = 3)	Middle ureter (*N* = 9)	Distal ureter (*N* = 24)	*P* value
Para-aortic lymph node	46 (73.0%)	22 (81.5%)	3 (100%)	8 (88.9%)	13 (54.2%)	0.099
Common iliac lymph node	20 (31.7%)	7 (25.9%)	2 (66.7%)	3 (33.3%)	8 (33.3%)	0.490
Internal iliac lymph node	4 (6.3%)	0 (0%)	0 (0%)	0 (0%)	4 (16.7%)	0.018
External iliac lymph node	5 (7.9%)	0 (0%)	0 (0%)	0 (0%)	4 (16.7%)	0.018
Renal fossa	6 (9.5%)	6 (22.2%)	0 (0%)	0 (0%)	0 (0%)	0.007
Ureter bed	16 (25.4%)	1 (3.7%)	2 (66.7%)	2 (22.2%)	11 (45.8%)	0.001

**Fig. 2:**
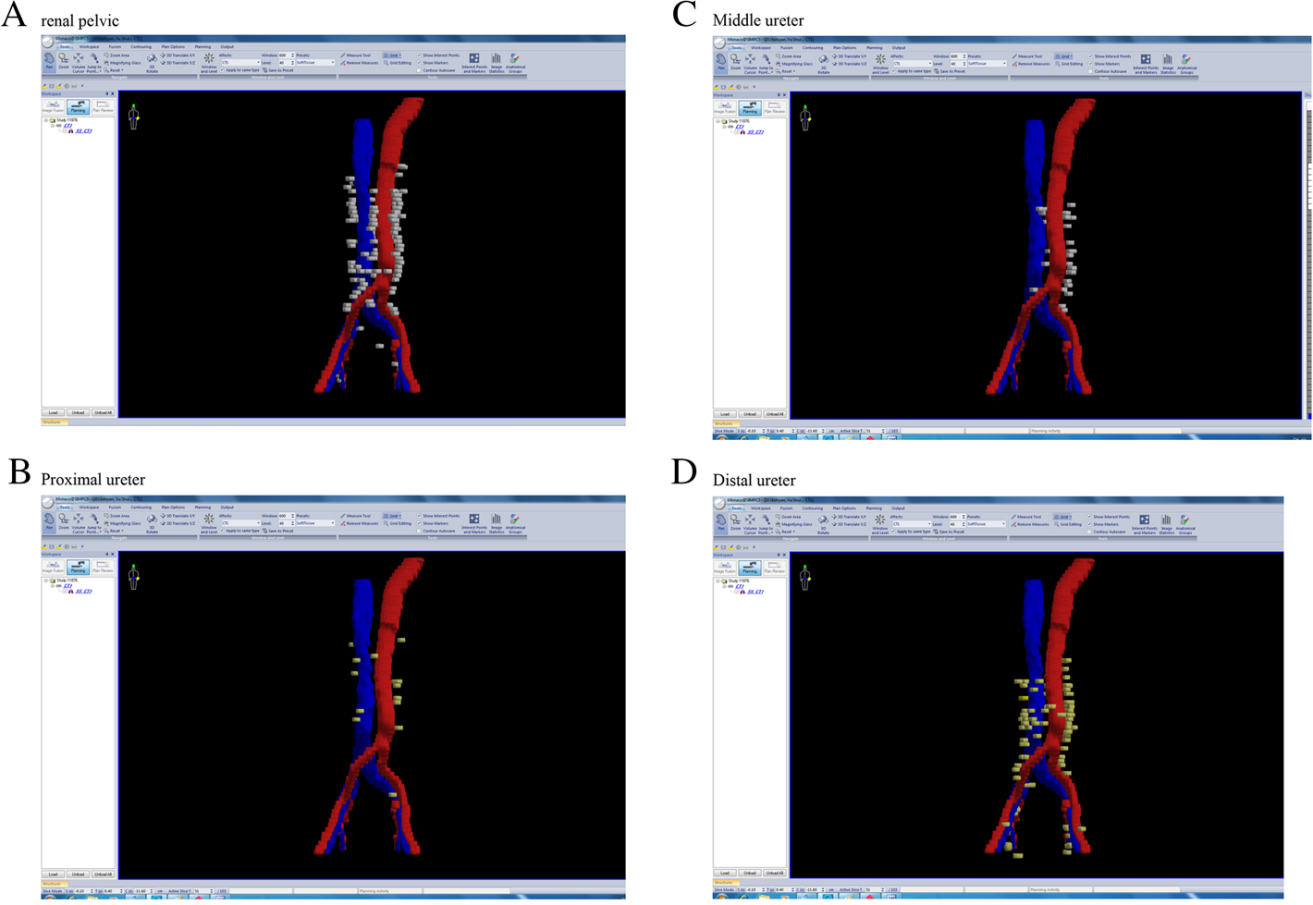
Recurrence lymph node distribution map stratified by primary tumor location. **a** Recurrence lymph nodes of pelvic tumor. **b** Recurrence lymph nodes of proximal ureter tumor. **c** Recurrence lymph nodes of middle ureter tumor. **d** Recurrence lymph nodes of distal ureter tumor (red, artery; blue, vein)

A total of 249 recurrent lymph nodes were recorded. Table 4 shows the distribution number of local recurrence lymph nodes. Most of the recurrent lymph nodes were in the para-aortic lymph node region (84.7%). In the renal pelvic group, more than 90% of all the recurrence lymph nodes were in the para-aortic region. In the proximal ureter group, the ratio was 88.9%. In the middle and distal ureter tumor groups, the recurrent para-aortic lymph node ratios decreased (84% and 75.3% respectively; Table 4). Thus, we plotted all the local recurrence lymph nodes on the CT image of a patient according to their relative positions to vessels and constructed a three-dimensional local recurrence map (Fig. 2 shows the local recurrence lymph node distribution stratified by primary tumor location).

**Table 4:** Local recurrence lymph node number of UTUC with different primary tumor locations

Local relapse region	All patients (*N* = 249)	Renal pelvic (*N* = 109)	Proximal ureter (*N* = 18)	Middle ureter (*N* = 25)	Distal ureter (*N* = 97)
Para-aortic lymph node	211 (84.7%)	101 (92.7%)	16 (88.9%)	21 (84.0%)	73 (75.3%)
Common iliac lymph node	28 (11.2%)	8 (7.3%)	2 (11.1%)	4 (16.0%)	14 (14.4%)
Internal iliac lymph node	4 (1.6%)	0 (0%)	0 (0%)	0 (0%)	4 (4.1%)
External iliac lymph node	6 (2.4%)	0 (0%)	0 (0%)	0 (0%)	6 (6.2%)

**Fig. 3:**
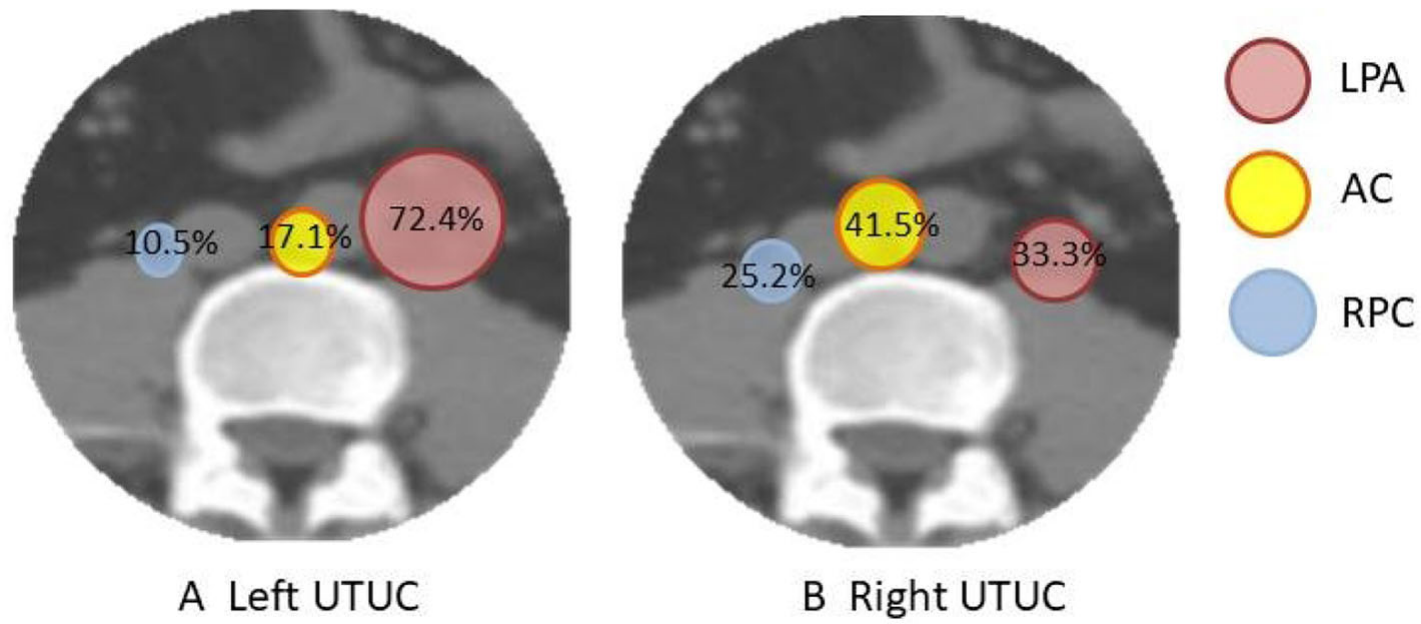
Recurrence lymph node distribution ratio stratified by primary tumor site. **a** Recurrence lymph node distribution ratio of left UTUC patients. **b** Recurrence lymph node distribution ratio of right UTUC patients (red, left para-aortic; yellow, aortocaval; blue, right paracaval)

We also investigated the features of para-aortic lymph node recurrence pattern for left- and right-side UTUC patients. Left-side tumor patients had totally 76 recurrent lymph nodes in the para-aortic region. Of these, 55 (72.4%) were located at LPA, 13 (17.1%) were located at AC, and 8 (10.5%) were located at RPC. Right-side tumor patients had 135 recurrent lymph nodes in the para-aortic region: 45 (33.3%) were located at LPA, 56 (41.5%) were located at AC, and 34 (25.2%) were located at RPC region. Our study found that most of the recurrent lymph nodes of left-sided UTUC patients occurred in the LPA region. For right-sided UTUC patients, recurrent lymph nodes in the para-aortic region were distributed in the LPA, AC, and RPC regions (Fig. 3).

## Discussion

RNU is the standard treatment of UTUC. The 5-year cancer-specific survival of early stage (≤ pT2N0) UTUC patients treated with RNU was fair (74.7%), the rates dramatically decreased in patients with advanced disease (pT3, 54.0% and pT4, 12.2%) or nodal involvement (35.5%) [25, 26].

Bladder, local, and distant recurrences were the major recurrence types after RNU [17]. Approximately 30% of patients with UTUC will have bladder recurrence (BR) [27, 28]. Series of studies have been done on the prognosis and risk factors of bladder recurrence after surgery [28, 29]. Our previous study found lower tumor grade, tumor multifocality, concomitant carcinoma in situ, and tumors located in the lower ureter were significant risk factors of bladder recurrence after RNU [27]. We also mapped the bladder recurrence sites of UTUC by primary tumor locations. We found most bladder recurrences occurred around the site of surgery and the posterior wall [30].

About 20-30% UTUC patients may have retroperitoneal and/or pelvis field relapse after RNU [17, 31]. In the advanced stage, the local recurrence rate may reach up to 65% [9]. T stage and multifocality were associated with increased local recurrence [6, 9, 31]. Effects of other factors such as incomplete surgery, venous invasion, G stage [31], and tumor locations [15] on local recurrence were debated. In UTUC, template-based lymphadenectomy appears to improve CSS and reduces the risk of local recurrence in patients with advanced tumor stage [16]. But nowadays, LND is not routinely performed during RNU [18, 32]. The effects of LND and LNM status on local recurrence were seldom analyzed in previous studies. In our study, the LND rate [6, 33] and extent of our lymphadenectomy [34] were consistent with other studies. It was interesting that patients who underwent LND had a higher local recurrence rate (34.2% vs 15.2%, *P* < 0.05) in our study. Based on previous literature, adequate lymph nodes removed should be more than eight [35]. But in our study, the median lymph node number removed was 4 (1–36). Only 27.4% (twenty of seventy-three) patients had their lymph nodes removed more than eight times. This indicated the LND extent and lymph node number removed nowadays were not enough.

Adjuvant radiotherapy may benefit patients with high local recurrence factors. However, there were relatively little clinical pieces of evidence supporting the efficacy of adjuvant radiotherapy for UTUC. The effects of adjuvant radiotherapy were argued in previous studies. Some studies found adjuvant radiation treatment had no benefit for local recurrence control [10, 36]. This may because they enrolled small and heterogeneous populations. For example, patients in radiotherapy group had a higher positive surgical margin rate in Huang’s study [36]. Thirty-three of 126 patients had residual tumor in Ozsahin’s study [10]. Other studies found adjuvant radiotherapy could improve local control in advanced stage UTUC patients. Jwa [37] found adjuvant radiotherapy could improve local recurrence-free survival, and Chen [38] found a benefit to adjuvant radiotherapy in overall survival. Recently, one study found in patents with pT3bN0-x UTUC, adjuvant radiotherapy alone was found to significantly reduce not only local recurrence but also distant metastasis and improve overall survival [39]. Their result suggested that adjuvant radiotherapy for local treatment might be helpful in controlling occult remnant diseases. The rationale of using adjuvant radiotherapy is to reduce local recurrence and potentially to halt distant disease progression and delay metastasis which may improve overall survival. Although guidelines have no suggestions of adjuvant radiotherapy. After recognizing the high local recurrence rate in advanced stage UTUC patients [9], our institutions have actively conducted adjuvant treatments such as radiotherapy or chemotherapy in patients with pT3, positive lymph node or G3 since 2011. Most of the patients refused to have adjuvant chemotherapy after surgery for fear of kidney function damage, some of them chose adjuvant radiotherapy. In our study, adjuvant radiotherapy group had more T3-4, G3, and LVI. These factors were all related with higher local recurrence in our study (Supplementary table). However, the local recurrence rate in the adjuvant radiotherapy group was lower. Adjuvant radiotherapy could reduce local recurrence rates even after multivariable analyses (*P* < 0.05). Our study also found patients with local recurrence had poor survival prognosis. Adjuvant radiotherapy may benefit high-risk patients with not only local control but also survival prognosis. Prospective study is needed on whether adjuvant radiotherapy can improve survival prognosis of patients with high-risk factors.

The precise understanding of local recurrence risk factors can assist clinicians in clinical risk stratification, adjuvant radiotherapy option, and surveillance arrangement. Efficacy of the postoperative radiation therapy depends on accurate delineation of clinical target volume to eradicate microscopic disease in the surgical bed while adjacent organs can be spared from high-dose irradiation with minimal toxicities. The upper urothelial tract is a large area; lymphatic drainage of the ureter and renal pelvis is different. The lymphatic of the renal pelvis drained along the renal vessels, whereas the lymphatic drainage of the ureter was segmented and diffuse [40]. For UTUC patients with template LND, UTUC with different primary site had different lymph node metastases patterns [12]. Lymph node metastasis rates in hilar and para-aortic region were higher for primary tumors of renal pelvic and proximal ureter. External and internal iliac lymph node metastasis rates were relatively higher for middle and distal ureter [12, 13]. Results from several small series suggested different patterns of local failure of UTUC based on location of the primary cancer in the upper urothelial tract [13, 15, 17, 41]. Tanaka found renal pelvic and proximal ureter patients had higher abdomen cavity local recurrence rates (88.2% and 90.9%, respectively). Distal (73.3%) and middle (36.8%) ureter patients had a higher prevalence of local recurrence rates in the pelvic cavity [17]. Surena also found upward migration of metastases to the paracaval and para-aortic regions from middle and distal ureteral tumors. Distal ureter tumors had lymph node metastases in external and internal region [13].

However, there are still no studies of the local recurrence map in UTUC patients stratified by tumor locations. Now, the clinical target volumes of UTUC patients received postoperative radiation are a wide area which includes the renal fossa, the course of the ureter to the entire bladder, and the paracaval and para-aortic lymph nodes. More than 50% of patients had acute gastrointestinal reaction side effects and hematological toxicity [37, 38]. Precise understanding of local recurrence pattern of UTUC can assist oncology clinicians to design a better radiation field in order to reduce the side effects of radiotherapy. This study provided a detailed picture of the local recurrence map of UTUC after RNU. Our results showed primary tumor locations were associated with unique patterns of local recurrence. We found the para-aortic lymph node region was the most common recurrence area for all the UTUC patients. Common iliac lymph node recurrence was the second high recurrence area. The recurrence rates in these two regions were almost the same for different primary tumor locations (*P* > 0.05). In our study, distal ureter tumor had a higher recurrence rate in the external and internal regions (*P* < 0.05). This result was consistent with the metastases pattern of UTUC.

UTUC had characteristic patterns of LNM dependent on the side and anatomic location of the primary tumor, including right to left migration [13]. For the right side, dissection of the hilum, paracaval, retrocaval, and interaortocaval region would capture nearly all metastasis lymph nodes. For the left side, a hilar and para-aortic dissection would capture nearly all primary metastasis lymph nodes [12, 13]. In our study, we found that the left-side tumor had most of the recurrent lymph nodes located in the LPA region. Recurrences in the AC and RPC regions were rare. Right-side tumor had recurrent lymph nodes distributed in the LPA, AC, and RPC regions. This result was consistent with the LNM pattern of UTUC.

Pelvic and ureter transitional carcinoma cell (TCC) are not the same disease in terms of invasion and prognosis. The renal pelvic tumor and adjacent tissue, which might contain micro-metastases, can be completely excised within a sufficient solid barrier. The ureter is only surrounded by weak adipose tissue making it more difficult to remove a ureteral tumor within sufficient surrounding tissues. Ureter TCC was associated with a higher local or distant failure rate than renal pelvic TCC [41]. Yoo found tumor location was related with local recurrence of UTUC after surgery [15]. In their study, the local recurrence for ureter tumor patients was almost 3 times of that in pelvic tumor patients (14.3% vs 5.2%).In our study, we also found the same trend, the renal pelvic tumor had a relatively lower local recurrence rate than ureter tumor (HR 0.625; *P* = 0.047 in univariable Cox regression analysis). Tumor bed recurrence rates were also different in pelvic and ureter tumors. Recurrence in renal fossa was only found in the renal pelvic tumor group. Ureter bed recurrence was more prevalent in ureter tumor. Whereas, patients in the renal pelvic rarely had ureter tumor bed recurrence (*P* < 0.05). Renal fossa recurrence rate of renal pelvic tumor was also lower than ureter tumor bed recurrence of ureter tumor (22.2% vs 45.8%). This result was similar with Yoo’s [15]. They found ureter tumor location was associated with surgical bed recurrence, the recurrence rate was almost 4 times of pelvic tumor (8.1% vs 2.1%, *P* = 0.011).

However, there were still some limitations to our study. First, this was a retrospective study. The definitive locations of recurrence sites were dependent on positive medical image scans taken during the follow-up period. Second, in this study, local recurrence sites were based on medical images, and there was no pathological verification of the recurrence sites.

## Conclusions

Our study for the first time illustrated the risk factors and site-specific local recurrence pattern stratified by tumor location in UTUC patients. To our understanding, this study is the first serry to map the local failure sites in patients with UTUC after radical surgery. Due to some limitations, there is a need to include more cases and to conduct a larger-sample prospective study in the future.

## Supplementary information

**Additional file 1: Supplementary Table.** Patient clinical and pathological characteristic

## Data Availability

The datasets used and/or analyzed during the current study are available from the corresponding author on reasonable request.

## Abbreviations

UTUC: Upper tract urothelial carcinoma
LVI: Lymphovascular invasion
PET: Positron emission tomography
RNU: Radical nephroureterectomy
TURBT: Transurethral resection of bladder tumor
LND: Lymph node dissection
LNM: Lymph node metastasis
TCC: Transitional carcinoma cell
LPA: Left para-aortic
AC: Aortocaval
RPC: Right paracaval
