# Supplementary material for: Pattern and risk factors of local recurrence after nephroureterectomy for upper tract urothelial carcinoma
Source: World J Surg Oncol. 2020 May 30;18:114. doi: 10.1186/s12957-020-01877-w (PMC7261378; doi:10.1186/s12957-020-01877-w)
Supplement: Supplementary file 1 — Additional file 1: Supplementary Table. Patient clinical and pathological characteristic [file 12957_2020_1877_MOESM1_ESM.docx]

**Supplement Table Patient clinical and pathological characteristic**

| Characteristics | Patients with RT(%) | Patients without RT (%) | P value |
| --- | --- | --- | --- |
| Patients number | 57 | 332 |  |
| Age group  <70  >=70 | 33(57.9%)  24(42.1%) | 169(50.9%)  163(49.1%) | 0.329 |
| Gender  Male  female | 30(52.6%)  27(47.4%) | 160(48.2%)  172(51.8%) | 0.536 |
| Site  Left  right | 29 (50.9%)  28(49.1%) | 176(53%)  156(47%) | 0.587 |
| Location  Pelvic  Ureter | 31(54.4%)  26(45.6%) | 151(45.4%)  181(54.5%) | 0.985 |
| Surgical procedure  Laparoscopic nephroureterectomy  Open nephroureterectomy | 53(93.0%)  4(7.0%) | 273(82.2%)  59(17.8%) | 0.077 |
| Lymph vascular invasion  Yes  No | 14(24.6%)  43(75.4%) | 44(13.3%)  288(86.7%) | 0.027 |
| Tumor grade  G1  G2  G3 | 0  19(33.3%)  35(61.4%) | 6(1.8%)  190(57.2%)  134(40.4%) | 0.000 |
| Pathological stage  T1  T2  T3  T4 | 2 (3.5%)  21(36.8%)  32(56.1%)  2(3.5%) | 123(37%)  99 (29.8%)  103(31%)  7(2.1%) | 0.000 |
| Lymph node detection  No  Yes | 43(75.4%)  14(24.6%) | 273(82.2%)  59(17.8%) | 0.225 |
| Lymph node involvement  Nx  N0  N1  N2 | 43(75.4%)  10(17.5%)  2(3.5%)  2(3.5%) | 273(82.2%)  33(9.9%)  9(2.7%)  17(5.1%) | 0.363 |
| Multifocality  No  Yes | 46(80.7% )  11(19.3%) | 275(82.8%)  57(17.2%) | 0.696 |
| Margin  Positive margin  Negative margin | 3(5.3%)  54(94.7%) | 9(2.7%)  323(97.3%) | 0.303 |
